# 

**Published:** 1885-04

**Authors:** 


PAGE.

ORIGINAL COMMUNICATIONS:

Permanganate of Potassium in Amenorrhœa. E. J. Doering, M. D. .... 283

The Influence of Cimicifuga Racemosa on Parturition. J. Suydam Knox, M. D. .... 287

Two Interesting Cases in Obstetrics. Charles Caldwell, M. D. .... 291

Chancre of the Eyelid and Caruncle. E. L. Holmes, M. D. .... 296

EDITORIAL:

Irresponsible Opinion .... 300

External Pelvic Measurement .... 301

Sanitary Inspection .... 304

Suicide .... 305

FOREIGN CORRESPONDENCE .... 307

SOCIETY PROCEEDINGS:

Chicago Medical Society. January 19th. The Bacillus of Syphilis. Josef Zeisler, M. D. .... 312

Counting a Rapid Pulse. A. E. Hoadley, M. D. 315

Obstruction of the Ileum by Peritoneal Adhesions. A. E. Hoadley, M. D. .... 315

Chicago Medical Society. February 2nd. Peroxide of Hydrogen in Aural and Ophthalmic Practice. B. Bettman, M. D. .... 318

Missed Abortion. P. O'Connell, M. D. .... 321

PAGE.

Muriate of Apomorphia in Chronic Bronchial Asthma. G. W. Webster, M. D. .... 323

Chicago Medical Society. February 16th. Malignant Disease of the Thyroid Gland. C. E. Webster, M. D. .... 324

Tape-Worm in a Child. C. G. Davis, M. D. .... 325

Hygroma of the Tongue. Josef Zeisler, M. D. .. 326

Comma Bacillus. L. L. McArthur, M. D. .... 327

Chicago Medical Society. March 2nd. Mollities Ossium. J. W. Webb, M. D. .... 327

Typhoid Fever. David O'Shea, M. D. .... 330

College of Physicians of Philadelphia. Meeting February 4th. Contagiousness of Tuberculosis. W. H. Webb, M. D. .... 334

CLINICAL REPORTS:

Medical Clinical Institute, Genoa, Italy. A. Lagorio, M. D. .... 365

BOOK REVIEWS .... 370

BOOKS RECEIVED .... 373

PAMPHLETS RECEIVED .... 375

American Medical Association .... 377

LOCAL NEWS:

Chicago Medical Society. Annual Election, etc. 377

PERSONAL:

Death of Professor J. L. Little, M. D., New York. 378

" " Abner Hard, M.D., Aurora .... 378

